# Robot-Assisted Parathyroidectomy Using Indocyanine Green (ICG) Fluorescence in Primary Hyperparathyroidism

**DOI:** 10.3390/medicina59081456

**Published:** 2023-08-12

**Authors:** Shin-Young Park, Yun Suk Choi, Young Mi Hwang, Jin Wook Yi

**Affiliations:** Department of Surgery, Inha University Hospital & College of Medicine, Incheon 22332, Republic of Korea

**Keywords:** primary hyperparathyroidism, robotic parathyroidectomy, indocyanine green fluorescence

## Abstract

*Background and Objectives*: Surgical treatment for primary hyperparathyroidism (PHPT) has evolved from bilateral exploration through a long transcervical incision to focused parathyroidectomy with a minimal incision above the pathologic gland. Recently, endoscopic or robot-assisted parathyroid surgery without direct neck incision has been introduced. The aim of this study was to investigate the effectiveness of indocyanine green (ICG) fluorescence as a new method for the visual identification of abnormal hyperfunctioning parathyroid glands in robot-assisted parathyroidectomy using Firefly^TM^ technology. We also aimed to conduct a comparative analysis between robot-assisted parathyroidectomy and conventional focused parathyroidectomy in order to identify clinical differences between the two surgical approaches. *Materials and Methods*: A total of 37 patients with PHPT underwent parathyroidectomy at a single university hospital between September 2018 and December 2022. Thirty-one patients underwent open focused parathyroidectomy (open group), and six patients underwent robot-assisted parathyroidectomy (robot group). Pre-operative localization via parathyroid SPECT-CT and an intraoperative parathyroid hormone (IOPTH) assay were used to successfully remove the pathologic parathyroid in both groups. ICG was administered only in the robot group. *Results*: Pathologic parathyroid showed a persistent fluorescence pattern under near-infrared vision. After the removal of the fluorescent parathyroid gland, IOPTH was normalized in all six patients in the robot group. However, the open group showed shorter hospital stays (1.8 ± 1.2 vs. 3.0 ± 0.0 days, *p* < 0.001) and shorter operation times (91.1 ± 69.1 vs. 152.5 ± 23.6 min, *p* = 0.001) than the robot group. After 6 months of surgery, PTH, calcium, and ionized calcium levels were all normalized without significant differences between the groups. *Conclusions*: Robot-assisted parathyroidectomy using ICG is helpful for the visual identification of the pathologic parathyroid gland. The advantage of robot parathyroidectomy is a better cosmetic outcome. However, it still does not show better clinical outcomes than conventional open focused parathyroidectomy.

## 1. Introduction

Primary hyperparathyroidism (PHPT) is a condition characterized by the overproduction of parathyroid hormone (PTH) from hyperfunctioning parathyroid glands. The typical clinical laboratory findings include elevated serum calcium levels and elevated PTH levels [1]. PHPT is more frequent in women, with an approximately 3~4:1 ratio in previous reports [2,3]. The incidence and prevalence of PHPT has increased in recent years due to the set order for various variables, including calcium and phosphorous levels, in many types of blood chemistry tests [4,5,6]. The incidence of PHPT in the United States was reported to be 66 cases per 100,000 person-years in women and 25 cases per 100,000 person-years in men [4].

PHPT is primarily caused by a solitary benign parathyroid adenoma, accounting for approximately 80% of patients. Multi-glandular disease is found in approximately 15–20% of PHPT patients and typically manifests as multi-gland parathyroid hyperplasia or, rarely, multiple parathyroid adenomas. Parathyroid cancer is extremely rare, accounting for less than 1% of all PHPT [7]. Most patients who are diagnosed with PHPT show a sporadic occurrence pattern, without any hereditary or family history of parathyroid-related disease or other multiple endocrine disorders [8,9].

The surgical removal of abnormal parathyroid glands is the definitive treatment for PHPT. The surgical approach in PHPT has evolved from the traditional transcervical bilateral exploration method through a 7 cm or longer incision on the neck to a more minimally invasive approach using a 2.5–3 cm skin incision, so-called focused parathyroidectomy. Focused parathyroidectomy can be achieved by the advancement of pre-operative imaging tests, including functional images of parathyroid SPECT-CT and high-resolution ultrasound, enabling the pre-operative localization of the pathologic parathyroid gland. Furthermore, the utilization of the intraoperative PTH (IOPTH) assay is very helpful to prompt the assessment of the successful removal of the pathologic parathyroid gland during surgery.

With the development of robotic head and neck surgery, the application of robotic surgery in parathyroidectomy has been proposed as a new parathyroidectomy method with favorable cosmetic outcomes. The utilization of near-infrared (NIR) fluorescence imaging in da-Vinci robot systems (Intuitive Surgical Inc., Sunnyvale, CA, USA), known as Firefly^TM^ technology (Novadaq Technologies Inc., Mississauga, ON, Canada), was proposed for the identification of parathyroid glands during thyroidectomy to preserve the parathyroid glands [10]. While previous studies mainly utilized ICG as a strategy to preserve parathyroid glands during thyroidectomy, we suggest that pathologic parathyroid glands also be identified via ICG fluorescence under the Firefly^TM^ function in robot surgery [11,12].

The aim of our study was to investigate the effectiveness of ICG fluorescence as a new method for the visual identification of abnormal hyperfunctioning parathyroid glands. We also aimed to perform a comparative analysis between robot-assisted parathyroidectomy and conventional focused parathyroidectomy to find the clinical difference between the two surgical approaches.

## 2. Materials and Methods

### 2.1. Patients

From September 2018 to December 2022, a total of 37 patients underwent parathyroidectomy due to PHPT in a single university hospital. All patients were included in the analysis without specific exclusion. Among the 37 patients, 31 patients underwent transcervical open parathyroidectomy (open group), and 6 patients received robot-assisted parathyroidectomy (robot group) using the bilateral axillary breast approach (BABA) [13]. We retrospectively reviewed the electronic medical records for the patients’ clinical information, laboratory results, surgical findings, hospitalization records, and pathologic reports.

### 2.2. Surgical Indication

Surgical indication of PHPT was followed by the National Institute for Health consensus guidelines as follows: patients with clinical symptoms of PHPT, such as nephrolithiasis, fractures, and symptomatic hypercalcemia; for asymptomatic PHPT patients, surgery was decided on if serum calcium levels exceeded 1.0 mg/dL above the normal range, creatinine clearance was less than 60 cc/min, the presence of nephrocalcinosis or nephrolithiasis was detected through radiologic findings, 24 h urine calcium excretion exceeded 400 mg/day, the presence of osteoporosis was indicated by a T score of −2.5 or lower at any site, a clinical fragility fracture was present, a vertebral compression fracture was present on spine imaging, their age was <50 years, or if there was the presence of clinical or biochemical evidence suggestive of parathyroid carcinoma [14].

### 2.3. Surgical Methods

All surgeries were performed by a single endocrine surgeon (JW Yi). Before the surgery, the location of the pathologic parathyroid gland was identified via parathyroid SPECT-CT and ultrasound. Intraoperative neuromonitoring (NIM 3.0, Medtronic, Minneapolis, MN, USA) was used in all surgeries. A 2–3 cm skin incision was used to perform open focused parathyroidectomy above the pre-operative skin marking for the pathologic parathyroid gland.

For robotic parathyroidectomy, the BABA method was used as reported elsewhere [13,15]. The patient was positioned in the supine posture with neck extension. Then, 8 mm skin incisions were made on the bilateral axilla and bilateral areola towards the neck, creating a subcutaneous flap using hydro-dissection with an advanced bipolar device. Robot docking was then performed through the axillary and breast ports. After robot docking, surgery was performed as follows: division of the midline strap muscle, thyroid gland mobilization without isthmectomy, dissection between the thyroid and strap muscles, identification of the pathologic parathyroid gland, identification of the recurrent laryngeal nerve, ICG injection and checking the intensity of the parathyroid gland, and resection of the parathyroid gland. All of the resected parathyroid was sent to pathologists to check the frozen biopsy, to confirm whether the resected organ was parathyroid or not.

### 2.4. ICG Angiography

For ICG angiography, a vial containing 25 mg of ICG was mixed with 10 mL of normal saline solution to obtain a 2.5 mg/mL concentration. Afterward, a syringe was used to extract 4 cc of the ICG solution from the vial, which corresponded to a dose of 10 mg of ICG for a 60 kg adult (0.17 mg/kg). This dose aligns with the safe range of ICG doses used for liver excretory function tests, where up to 30 mg of ICG is considered acceptable for a 60 kg adult. After the strap muscle was dissected from the thyroid, the ICG solution was administered intravenously. Following the injection, the Firefly^TM^ system was activated to enable fluorescence imaging [11].

### 2.5. IOPTH Monitoring

The IOPTH assay was conducted at four specific time intervals during the surgery: pre-incision, pre-excision, and at 5 min and 10 min after removing the diseased parathyroid gland. In our institution, the Miami criterion was used to determine the successful removal of the hyperfunctioning parathyroid gland in both focused and subtotal parathyroidectomy procedures. According to this criterion, a >50% decrease in PTH levels 10 min after parathyroidectomy, compared to the highest level of PTH before the excision, was considered indicative of a successful procedure. If the PTH level did not decrease successfully in focused parathyroidectomy, bilateral exploration was indicated.

### 2.6. Statistics and Ethical Consideration

For continuous variables, unpaired *t*-tests were used for comparison. Categorical variables were compared using the Chi-square or Fisher’s exact test according to sample size. A *p* value under 0.05 was considered indicative of statistical significance. All statistical analyses were carried out using version 4.3.0 of the R programming language (R: A language and environment for statistical computing. Vienna, Austria. https://www.R-project.org/ (accessed on 4 June 2023)). The ethics of this study were approved by the institutional review board in the author’s hospital (INHAUH 2023-04-026).

## 3. Results

Table 1 shows the patients’ clinical and pathologic characteristics. The mean age of all patients was 56.1 ± 12.6 years, with 29 female patients and 8 male patients. Among the 37 patients, 34 underwent one-gland parathyroidectomy, 2 underwent two-gland parathyroidectomy, and 1 underwent bilateral exploration due to the vague location of the pathologic parathyroid glands. The most frequently affected gland locations were identified as the right lower region (15 glands) and the left lower region (12 glands). The pathologic diagnosis revealed that 28 patients had parathyroid adenoma, while 11 patients had parathyroid hyperplasia. In terms of pre-operative imaging tests, all patients underwent ultrasound. SPECT-CT was performed in all patients except one. The most common clinical manifestations were ureter or renal stones (14 patients) and osteoporosis (9 patients).

**Figure 1:**
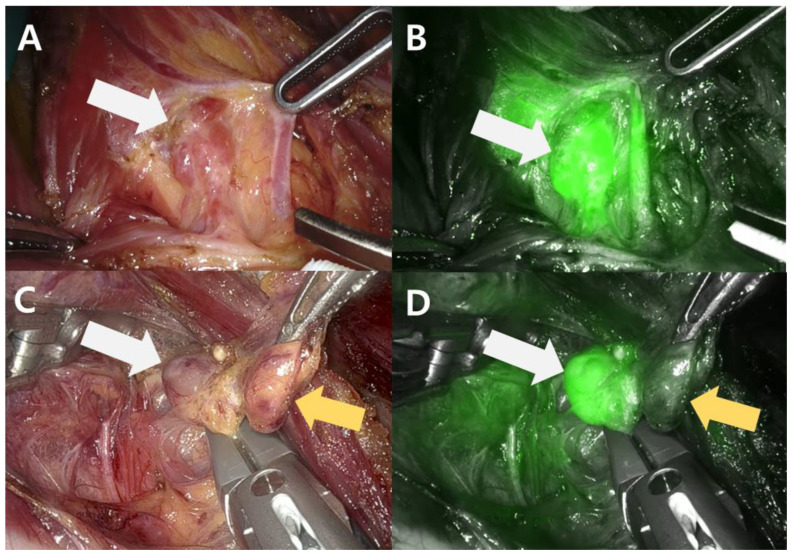
Parathyroid view in robot BABA parathyroidectomy. (**A**) Right parathyroid under natural vision; (**B**) ICG fluorescence imaging of the right parathyroid under NIR; (**C**) left parathyroid under natural vision; (**D**) ICG fluorescence imaging of the left parathyroid under NIR. The white arrows indicate the parathyroid glands, and the yellow arrows indicate the lymph nodes. The parathyroid gland exhibited fluorescence under NIR light, whereas the lymph node did not, allowing for differentiation between them despite their similar appearance.

In the robot group using ICG, the parathyroid gland started to be enhanced after approximately 3 min, as shown in Figure 1. The pathologic parathyroid gland showed a notable enhancement pattern before thyroid gland enhancement and persistent fluorescence, as shown in Figure 1A,B. Unlike the parathyroid gland, lymph nodes that looked similar to the parathyroid gland did not show ICG uptake, as shown in Figure 1C,D. This ICG fluorescent pattern was observed in all six patients in the robot group. Frozen biopsy confirmed that the ICG fluorescent masses were parathyroid lesions.

Table 2 shows the comparative analysis between the open and robot group for the clinical and laboratory findings. Age, sex distribution, and BMI were not significantly different between the two groups. The operation time was shorter in the open group than in the robot group (91.1 ± 69.1 vs. 152.5 ± 23.6 min, *p* = 0.001). Estimated blood loss, largest gland size, and postoperative complications were not significantly different. Hospital stay days in the open group were shorter than those in the robot group (1.8 ± 1.2 vs. 3.0 ± 0.0 days, *p*< 0.001).

During the intraoperative PTH monitoring, there were no significant differences between the two groups in PTH levels at all time points. In all patients, a significant PTH decrease of more than 50% was observed 10 min after parathyroid excision. The postoperative calcium and ionized calcium levels were significantly lower in the open group (8.8 ± 0.7 vs. 10.3 ± 0.7 mg/dL, *p* < 0.001; 1.1 ± 0.1 vs. 1.4 ± 0.2 mmol/L, *p* < 0.001, respectively). However, no significant differences were observed in terms of calcium and ionized calcium levels between the two groups 6 months and 12 months after surgery.

**Figure 2:**
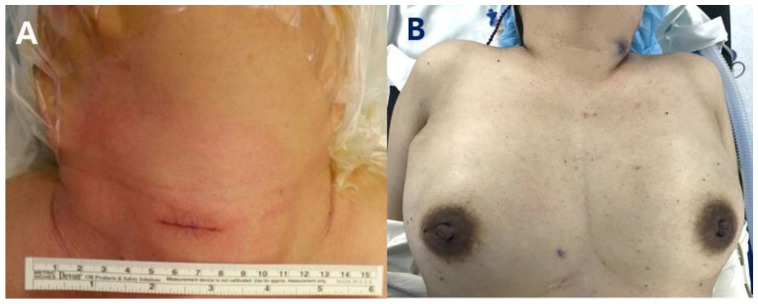
Postoperative scar after parathyroidectomy. (**A**) Focused parathyroidectomy made a minimal incision size of 2.5 to 3 cm on the neck; (**B**) a near absence of visible scarring can be observed in robot BABA parathyroidectomy.

Figure 2 shows the difference in postoperative scars between the two groups in photographs taken at the immediate postoperative time. The scar from open focused parathyroidectomy was smaller than that from traditional surgery, but it was still noticeable on the neck. On the other hand, in robot surgery, surgery scars were not visible, especially on the neck.

## 4. Discussion

In 1925, Doctor Felix Mandl performed the first parathyroidectomy for a patient who suffered from bone disease. He removed an enlarged parathyroid gland using the conventional transcervical open approach. Postoperatively, the patient’s calcium levels significantly decreased, and although there was gradual improvement in the bone disease over time, the disease regrettably resurfaced after 6 years [16]. Through accumulated experience, surgeons discovered that not all patients had solitary adenomas and realized the need to explore all four parathyroid glands to exclude multi-glandular disease. As a result, bilateral neck exploration has become the standard surgical approach for hyperparathyroidism, typically performed through a 7 cm or longer incision on the anterior neck.

The development of 99mTc-sestamibi in 1989 increased both the utility and sensitivity of nuclear imaging in HPT [17]. The use of 99mTc-sestamibi scans and neck ultrasounds could enhance the anatomical localization of diseased parathyroid glands, with individual accuracies of approximately 77% to 88% and 60% to 77%, respectively, in cases of sporadic PHPT. When both tests are used in combination, the accuracy increases significantly, ranging from 89% to 98% [18,19,20]. Pre-operative localization imaging plays an important role in the efficiency of the procedure and selection of patients for focused versus bilateral neck exploration. The IOPTH assay, which was first described by Nussbaum et al. in 1988, has been known to be highly beneficial in confirming the successful removal of the hyperfunctioning parathyroid gland during surgery [21]. The utilization of localization imaging and IOPTH assays has enabled the successful accomplishment of minimally invasive focused parathyroidectomy, which is performed through a minimal incision size of 2.5 to 3 cm on the neck [22,23,24].

Recently, the advancement of robotic surgery has facilitated the implementation of robotic systems in parathyroid surgery, enabling more precise and minimally invasive procedures while avoiding the formation of scars on the neck. Table 3 describes the current reports about robot parathyroidectomy. In reported robotic parathyroidectomy studies, the most common approach used was the transaxillary approach. The robot BABA approach was reported by He et al. in 2015, with no reported complications or cases requiring open conversion [25]. Among the various approaches, the complication rate was acceptable, and open conversion was rare, except for the lateral cervical approach. The use of real-time ICG imaging was only reported by Mohsin et al. in one case [26].

Through pre-operative imaging tests, the approximate location of the diseased parathyroid gland is identified. However, during the surgical procedure, the precise location of the parathyroid gland can be obscured and potentially confused by surrounding structures. ICG fluorescence imaging under NIR is a useful method that can help surgeons identify anatomical structures in various types of surgery in real time, such as the liver, common bile duct, and vascular structure. ICG is a water-soluble, tricarbocyanine dye that, when injected intravenously, rapidly binds to plasma proteins and exhibits fluorescence when excited with either a laser beam or NIR light with a wavelength of 820 nm and longer [39]. Sound et al. documented three successful cases of reoperative parathyroid surgery using video-assisted technique with ICG fluorescence imaging in 2015 [40]. The patients were given intravenous ICG at a total dose of 8.75 or 10 mg through two separate injections. The parathyroid glands became visible within 2 min after the injection, and the adenomas exhibited fluorescence for approximately 20 min. Zaidi et al. conducted the first prospective case study using intraoperative ICG fluorescent imaging of parathyroid glands, involving 33 patients who underwent open parathyroidectomy for PHPT. A dose of 5 mg of ICG was administered intravenously following adequate exposure of each central neck compartment. Despite the demonstrated uptake of ICG in parathyroid glands exceeding 90%, the practical application of ICG to guide the course of parathyroid exploration was limited due to the concurrent uptake of ICG by the thyroid gland [41]. In 2017, Yu et al. reported the results of using ICG under NIR for the effective identification and preservation of the parathyroid glands during BABA robotic thyroidectomy [11]. They recommended 10 mg of ICG as the optimal dose for a 60 kg adult (0.17 mg/kg). They reported that lower doses than 10 mg required a longer time for the fluorescence of the parathyroid to be observed after injection and resulted in weaker fluorescence intensity. They also reported that at this 10 mg dose, ICG fluorescence of the parathyroid glands could be observed, on average, 203 s after injection, which was approximately 4 s earlier than the onset of fluorescence in the thyroid glands. This time difference provided a sufficient interval to differentiate the parathyroid gland from the thyroid. We adopted the ICG dose used in the study from Yu et al. in our research.

In our study, pathologic parathyroid glands were all identified under ICG vision. All glands that showed ICG uptake were confirmed by pathologists as pathologic parathyroid glands. After resection of the pathologic parathyroid gland, a significant reduction of over 50% in PTH levels was achieved over 10 min in all robot surgery patients. We suggest that the advantages of Firefly^TM^ in the da-Vinci system using ICG injection in parathyroid surgery are as follows: visual identification of the pathologic parathyroid gland and distinguishing the parathyroid gland from adjacent lymph nodes, which presented a similar appearance under natural vision, as shown in Figure 1C,D. Furthermore, surgical scars are not visible in the neck area if bilateral parathyroid exploration occurs because the surgeon can approach all four parathyroid glands without changing the initial incision or making an additional incision that is required in open bilateral exploration.

After 6 months of surgery, PTH, calcium, and ionized calcium levels were all normalized and maintained in a normal state at the 1-year mark, with no significant differences observed between the two groups. These findings indicate that successful surgeries were achieved without significant postoperative complications, not only in the open group but also in the robot group.

However, the operation time was significantly longer in the robot group (91.1 ± 69.1 vs. 152.5 ± 23.6 min, *p* = 0.001). The reason for the longer operation time in the robot group compared to the open group is that in remote access surgeries such as robot BABA parathyroidectomy, there is a longer distance from the incision to the parathyroid gland compared to open surgery, and additional time is required for flap creation to reach the parathyroid gland. The length of hospital stay in the robot group was also significantly longer than that in the open group (1.8 ± 1.2 vs. 3.0 ± 0.0 days, *p* < 0.001). The reason for the shorter hospital stay in the open group compared to the robot group is that in patients undergoing open focused parathyroidectomy, the extent of flap creation is relatively smaller compared to robot-assisted procedures, resulting in a higher likelihood of not requiring a drain. However, considering that there were no significant differences in postoperative complications between the two groups and that patients can also be discharged with a drain, the difference in hospital stay is not considered to have clinically significant meaning. Additionally, the postoperative hospital stay of 3 days for the robot group itself is not excessively long.

One of the strengths of this study is its emphasis on the use of ICG for the easy detection and successful removal of diseased parathyroid glands, whereas many previous studies primarily focused on the use of ICG for preserving parathyroid glands in thyroid surgery [11,12,42,43,44,45,46,47,48,49,50,51]. To our knowledge, we have reported in this study the largest number of cases of robot BABA parathyroidectomy using ICG. Furthermore, this is the first study to compare robot parathyroidectomy using ICG with open parathyroidectomy. In our study, robot parathyroidectomy using ICG demonstrated noninferiority compared to open parathyroidectomy in terms of surgical outcomes and complications.

One limitation of this study is that all surgeries were performed by a single experienced surgeon in a single institution. To generalize the findings, it is necessary to include results from multiple surgeons, including less experienced surgeons, across various institutions. Another limitation is the relatively small sample size in this study. In particular, the number of cases in the robot group is insufficient to obtain statistically significant results. Including more patients through multi-institutional studies will contribute to obtaining generalized results and achieving statistically significant findings. Additionally, due to the low incidence rate of PHPT, it is necessary to include a larger number of patients in research through multi-institutional studies [5]. This study was a retrospective comparative study. Further prospective studies, including many patients, are required to establish clinically significant evidence.

## 5. Conclusions

Robot-assisted parathyroidectomy using ICG is a feasible and effective procedure for PHPT patients without significant complications. Further studies are needed to validate these findings.

## Figures and Tables

**Table 1:** Patient characteristics for primary hyperparathyroidism (*n* = 37).

Variables	Number of Patients
Age (years, mean ± sd)	56.1 ± 12.6
Sex	
Female	29
Male	8
BMI (kg/m^2^, mean ± sd)	23.9 ± 4.2
Parathyroidectomy extent	
One-gland parathyroidectomy	34
Two-gland parathyroidectomy	2
Bilateral exploration	1
Pathologic gland location	
Right upper	6
Right lower	15
Left upper	6
Left lower	12
Pathologic diagnosis	
Parathyroid adenoma	28
Parathyroid hyperplasia	11
Pre-operative imaging	
SPECT-CT	36
US	37
Comorbidity	
Hypertension	12
Chronic renal failure	4
Arrhythmia	2
Coronary artery disease	2
Osteoporosis	9
Fracture history	1
Ureter or renal stone	14

**Table 2:** Comparative analysis between open and robot parathyroidectomy.

Variables	Open (*n* = 31)	Robot (*n* = 6)	*p* Value
Age (years)	57.3 ± 12.6	49.7 ± 11.2	0.178
Sex			1.000
Female	24	5	
Male	7	1	
BMI (kg/m^2^, mean ± sd)	24.0 ± 4.4	23.7 ± 3.6	0.862
Operation time (min)	91.1 ± 69.1	152.5 ± 23.6	0.001
Estimated blood loss (mL)	46.1 ± 178.3	8.3 ± 20.4	0.262
Largest gland size (cm)	1.8 ± 0.9	1.7 ± 1.0	0.725
Postoperative complications (*n*)			1.000
Transient vocal cord palsy	1	0	
Permanent vocal cord palsy	0	0	
Hypertrophic scar or keloid	1	0	
Hospital stay days after surgery (days)	1.8 ± 1.2	3.0 ± 0.0	<0.001
PTH, pre-operative (pg/mL)	167.5 ± 95.7	443.8 ± 459.4	0.201
PTH, pre-incision (pg/mL)	207.9 ± 170.5	542.5 ± 400.9	0.097
PTH, pre-excision (pg/mL)	153.1 ± 81.4	720.5 ± 626.8	0.077
PTH, 5 min after excision (pg/mL)	76.6 ± 52.8	228.5 ± 207.5	0.133
PTH, 10 min after excision (pg/mL)	44.7 ± 26.1	160.3 ± 138.9	0.097
PTH, 6 months after surgery (pg/mL)	40.2 ± 18.5	29.7 ± 7.7	0.278
PTH, 12 months after surgery (pg/mL)	46.7 ± 27.5	30.0 ± 14.4	0.337
Calcium, pre-operative (mg/dL)	10.8 ± 0.7	12.7 ± 1.8	0.071
Calcium, postoperative (mg/dL)	8.8 ± 0.7	10.3 ± 0.7	<0.001
Calcium, 6 months after surgery (mg/dL)	9.3 ± 0.4	9.4 ± 0.3	0.673
Calcium, 12 months after surgery (mg/dL)	9.2 ± 0.5	9.2 ± 0.4	0.824
Ionized calcium, pre-operative (mmol/L)	1.4 ± 0.1	1.6 ± 0.3	0.095
Ionized calcium, postoperative (mmol/L)	1.1 ± 0.1	1.4 ± 0.2	<0.001
Ionized calcium, 6 months after surgery (mmol/L)	1.2 ± 0.1	1.2 ± 0.2	0.455
Ionized calcium, 12 months after surgery (mmol/L)	1.3 ± 0.2	1.3 ± 0.0	0.976

**Table 3:** Current literature on robot parathyroidectomy.

Year	Author	Study Design	Type of Approach	Patients (*n*)	Operation Time (min)	IOPTH	ICG	Complication	Conversion
2011	Landry [27]	Case series	Trans -axillary	2	108.5	Yes	No	No	No
2012	Foley [28]	Comparative	Trans -axillary	4	186	Yes	No	Yes (1 wound infection, 1 seroma)	No
2013	Boccara [29]	Case series	Trans -axillary	2	150	No	No	No	Yes (*n* = 1)
2014	Noureldine [30]	Case series	Trans -axillary	9	119	Yes	No	No	Yes (*n* = 1)
2014	Al Kadah [31]	Case series	Trans -axillary	2	N/A	No	No	No	No
2014	Karagkounis [32]	Case series	Trans -axillary	8	184	Yes	No	Yes (1 seroma)	No
2015	Tolley [33]	Comparative	Axillary and anterior chest	15	119	No	No	No	Yes (*n* = 1)
2015	He [25]	Case series	BABA	6	156	Yes	No	No	No
2017	Alshehri [34]	Case series	Retro -auricular	3	167.1	No	No	N/A	No
2017	Mohsin [26]	Case report	Trans -axillary	1	N/A	Yes	Yes	No	No
2018	Wu [35]	Case series	Trans -axillary	2	122.5	No	No	Yes (1 transient hypocalcemia)	No
2018	Ozdenkaya [36]	Case series	Transoral	4	184.7	Yes	No	No	Yes (*n* = 2)
2019	Slycke [37]	Case series	Lateral cervical	23	88	Yes	No	No	Yes (*n* = 9)
2021	Kandil [38]	Case series	Trans -axillary	102	116	Yes	No	Yes (1 wound infection, 1 seroma)	Yes (*n* = 1)

## Data Availability

No new data were created or analyzed in this study. Data sharing is not applicable to this article.
